# Second-order TGV model for Poisson noise image restoration

**DOI:** 10.1186/s40064-016-2929-3

**Published:** 2016-08-05

**Authors:** Hou-biao Li, Jun-yan Wang, Hong-xia Dou

**Affiliations:** School of Mathematical Sciences, University of Electronic Science and Technology of China, Chengdu, 611731 People’s Republic of China

**Keywords:** Image restoration, Poisson noise, Total generalized variation, Split Bregman iteration, Optimization problem

## Abstract

Restoring Poissonian noise images have drawn a lot of attention in recent years. There are many regularization methods to solve this problem and one of the most famous methods is the total variation model. In this paper, by adding a quadratic regularization on TGV regularization part, a new image restoration model is proposed based on second-order total generalized variation regularization. Then the split Bregman iteration algorithm was used to solve this new model. The experimental results show that the proposed model and algorithm can deal with Poisson image restoration problem well. What’s more, the restoration model performance is significantly improved both in visual effect and objective evaluation indexes.

## Background

Image restoration is a fundamental and unavoidable problem in digital image processing by reconstructing the blurred or noisy image from the observed image and not losing the details of image (Zhao et al. 2013; Bilal et al. 2015). There are abundant methods to restore these images. For example, in Zhao et al. (2013), a Bayesian minimum mean squared error (MMSE) estimation based on high-order non-local range-Markov random field (NLRMRF) prior is proposed for non-blind image deblurring problem. The constrained optimization framework (Bilal et al. 2015) was presented to solve the image spatial degradation problem. Moreover, an adaptive weighted regularization scheme was also proposed in modified error estimate (MEE) to cater with the uncertainty due to ill-posed nature of the inverse problem in Bilal et al. (2015). In terms of regularization models, one of the most remarkable models was the ROF model introduced by Rudin et al. (1992), which presented total variation regularization for removing the additive Gaussian white noise. The total variation (TV) model assumed the original image *u* to be defined on $\Omega \subset {{\mathbb {R}}^2}$ and obtained a solution from a minimization problem

1 $$\begin{aligned} F(u) := \int _\Omega {\left| {\nabla u} \right| } + \frac{\lambda }{2}{\int _\Omega {\left| {f - u} \right| } ^2}, \end{aligned}$$

where the first term is the total variation regularization term, the second term is fidelity term and $\lambda$ is a positive fidelity parameter. Although the TV model is effective in image processing, specially for Gaussian white noise. However, this model is not effective in restoring Poisson noise images such as astronomical (Starck and Murtagh 2007), biomedical (Dey et al. 2006; Sarder and Nehorai 2006; Willett and Nowak 2003), and photographic imaging (Foi et al. 2005). For this reason, scholars have proposed some new methods to deal with Poisson noise image problems.

Le et al. (2007) proposed a new total variation model to recover image corrupted by Poisson noise, the new total variation model with fidelity term is suitable for Poisson noise. The new model can be written as

2 $$\begin{aligned} \mathop {\min }\limits _u \int _\Omega {\left| {Du} \right| } + \lambda \int _\Omega {(Ku - f\log Ku)}. \end{aligned}$$

The authors used the gradient descent method to obtain its optimum solution. However, this method can not obtain optimal approximation when the image is both high intensity noise and low intensity features. In Sawatzky et al. (2013), an efficient EM-TV algorithm is presented to speed the computation of the optimization problem (2). In addition, alternating split Bregman iterative algorithm (Setzer et al. 2010) is also used to solve the question (2), since this algorithm does not contain iterations and also not produce negative values. In addition, Figueiredo and Bioucas-Dias (2010) proposed an approach based on alternating direction optimization method for deconvolving Poissonian images.

Recently, based on Chavent and Kunisch (1997) and Liu and Huang (2012) proposed another new total bounded variation-based Poissonian images restoration model

3 $$\begin{aligned} \mathop {\min }\limits _u \int _\Omega {\left| {Du} \right| }+\frac{{{\lambda_1}}}{2}\left\| u \right\| ^2 + \lambda_2 \int _\Omega {(Ku - f\log Ku)}. \end{aligned}$$

Although the above model is better than the total variation model and has a competitive superiority, there also exist some shortcomings, for example, sometimes it will cause undesired oil painting artifacts. In order to avoid the staircase effect, many methods have been proposed. A well-known method to eliminate staircase effect is the TGV (Bredies et al. 2010; Bredies and Valkonen 2011; Bredies et al. 2013) regularization. The TGV regularizer can effectively eliminate the staircase effect but there are still some shortcomings, it tends to introduce some blurring on image edges and texture regions as the existence of high-order derivative term. More seriously, some small details will be lost during the denoising. For this reason, we consider combine the TGV and $\left\| u \right\| ^2$ as one regularization term to solve the poisson noise image restoration problem.

The rest of this article is organized as follows. In “Total generalized variation (TGV)” section, we briefly review the total generalized variation (TGV). The proposed model and algorithm are presented in “The proposed Poisson noise recovering model and algorithm” section. In “Experimental results and discussions” section, experimental results are illustrated to show the consistent performance of the proposed method. Finally, conclusions are given in “Conclusions” section.

## Total generalized variation (TGV)

 Bredies et al. (2010) proposed the concept of total generalized variation (TGV), which is considered to be the generalization of TV. For convenience, some concepts of TGV are given as follows.

### **Definition 1**

(Bredies et al. 2010) Let $\Omega \subset {{\mathbb {R}}^d}$ be a domain, $k \ge 1$ and $\alpha=({\alpha _0}, \ldots ,{\alpha _{k - 1}} )> 0$. Then, the total generalized variation of order *k* with weight $\alpha$ for $u\in L_{loc}^1(\Omega )$ is defined as the value of the function

4 $$\begin{aligned} TGV_\alpha ^k(u)& = {} \sup \left\{ {\int _\Omega {udi{v^k}\nu dx|\nu \in } C_c^k\left( \Omega ,Sy{m^k}({{\mathbb {R}}^d})\right) ,} \right. \nonumber \\&\qquad \quad \left. { {{\left\| {di{v^l}\nu } \right\| }_\infty } \le {\alpha _l}, \quad l=0,\ldots , k-1} \right\} , \end{aligned}$$

where $Sym^k({\mathbb {R}}^d)$ denotes the space of symmetric tensors of order *k* with arguments in ${\mathbb {R}}^d$, and ${\alpha _l}$ are fixed positive parameters.

### **Definition 2**

(Bredies et al. 2010) The space of bounded generalized variation is defined as

5 $$\begin{aligned} \begin{aligned} BG{V^k_\alpha}(\Omega )&= \left\{ {u \in {L^1}(\Omega )|TGV_\alpha ^k(u) < \infty } \right\} ,\\ {\left\| u \right\| _{BG{V^k_\alpha}}}&= {\left\| u \right\| _1} + TGV_\alpha ^k(u). \end{aligned} \end{aligned}$$

Here $BG{V^k_\alpha}(\Omega )$ is a Banach space independent of the weight vector $\alpha$.

### **Definition 3**

(Bredies et al. 2010) The “dualization” in the definition of the functional $TGV_\alpha ^k$ can also be informally interpreted in terms of iterated Fenchel duality.

6 $$\begin{aligned} TGV_\alpha ^k(u) = \mathop {\mathop {\inf }\limits _{{u_l} \in {C^{k - l}}(\overline{\Omega },Sy{m^l}({{\mathbb {R}}^d}))}} \limits _{l= 1, \ldots ,k - 1,{u_0} = u,{u_k} = 0} \sum \limits _{l = 1}^k {{\alpha _{k - l}}{{\left\| {\varepsilon ({u_{l - 1}}) - {u_l}} \right\| }_1}}. \end{aligned}$$

Note that the tensor field ${u_l}$ are in different spaces for varying *l*. Moreover, the operator $\varepsilon \left( {{u_{l - 1}}} \right)$ denotes the symmetrized gradient operator

7 $$\begin{aligned} \varepsilon \left( {{u_{l - 1}}} \right) = \frac{{\nabla {u_{l - 1}} + {{\left( {\nabla {u_{l - 1}}} \right) }^T}}}{2}. \end{aligned}$$

In this paper, we use $k=2$ in the proposed model. Thus, the second-order TGV can be written as

8 $$\begin{aligned} TGV_\alpha ^2(u) = {} \sup \left\{ {\int _\Omega {udi{v^2}wdx|w \in C_c^2\left( \Omega ,{S^{d \times d}}\right) ,} }\; {{{\left\| w \right\| }_\infty } \le {\alpha _0},{{\left\| {div \; w} \right\| }_\infty } \le {\alpha _1}} \right\} , \end{aligned}$$

where ${S^{d\times d}}$ denotes the space of symmetric ${d \times d}$ matrices. And the first and second divergences are defined as

9 $$\begin{aligned} {(div \; w)_h}& = {} \sum \limits _{j = 1}^d {\frac{{\partial {w_{hj}}}}{{\partial {x_j}}}}, \quad 1 \le h \le d,\end{aligned}$$

10 $$\begin{aligned} di{v^2}w& = {} \sum \limits _{h,j = 1}^d {\frac{{{\partial ^2}{w_{hj}}}}{{\partial {x_h}\partial {x_j}}}}. \end{aligned}$$

In addition, according to Bredies and Valkonen (2011), the energy term $TGV_\alpha ^2$ can be formulated as

11 $$\begin{aligned} TGV_\alpha ^2 = \mathop {\min }\limits _{u \in BGV_\alpha ^2\left( \Omega \right) ,p \in BD\left( \Omega \right) } {\alpha _1}\int _\Omega {\left| {\nabla u - p} \right| } + {\alpha _0}\int _\Omega {\left| {\varepsilon \left( p \right) } \right| }, \end{aligned}$$

where $\varepsilon (p)$ can be separately expressed as

12 $$\begin{aligned} \varepsilon (p) = \left[ {\begin{array}{*{20}{c}} {{\nabla _x}{p_1}}&{}\quad {\frac{1}{2}({\nabla _y}{p_1} + \nabla {}_x{p_2})}\\ {\frac{1}{2}({\nabla _y}{p_1} + \nabla {}_x{p_2})}&{}\quad {{\nabla _y}{p_2}} \end{array}} \right] . \end{aligned}$$

## The proposed Poisson noise recovering model and algorithm

### The proposed model for Poisson noise image

We assume that $u \in {\mathbb {R}}_ + ^N$ is the original image, $f \in {{\mathbb {R}}^N}$ is the observed image, $K \in {{\mathbb {R}}^{N \times N}}$ is a linear blurring operator related with the spread point function (PSF). Then the degradation model can be described as

13 $$\begin{aligned} f=\mathrm{P} (Ku), \end{aligned}$$

where *P* denotes the Poisson distribution function.

Based on Le et al. (2007), we define the Bayes Law as follows.

14 $$\begin{aligned} P\left( {u|f} \right) = \frac{{P\left( {f|u} \right) P\left( u \right) }}{{P\left( f \right) }}. \end{aligned}$$

According to (14), for each $u\in \Omega$, we have

15 $$\begin{aligned} P(f|Ku) = \prod \limits _{i = 1}^N {P({f_i}|{{(Ku)}_i})} = \prod \limits _{i = 1}^N {\frac{{{e^{ - {{(Ku)}_i}}}{{({{(Ku)}_i})}^{{f_i}}}}}{{{f_i}!}}}. \end{aligned}$$

Next, we assume that the prior distribution *P*(*u*) is TGV and $\left\| u \right\| ^2$, which can be written as

16 $$\begin{aligned} p\left( u \right) = \exp \left( -\frac{{{\lambda }}}{2}\left\| u \right\| _2^2- TGV_\alpha ^2 \right) , \end{aligned}$$

where $\lambda$ is the regularization parameter.

Thus, we obtain a model for restoring the Poissonian noise image as follows.

17 $$\begin{aligned} \mathop {\min }\limits _u \beta \sum \limits _{i = 1}^N {{{(Ku)}_i} - {f_i}\log {{(Ku)}_i}} +\frac{{{\lambda }}}{2}\left\| u \right\| _2^2 + TGV_\alpha ^2 + {l_{{{\mathbb {R}}^ + }}}(u), \end{aligned}$$

where $l_s$ is the indicator function of set *S*

18 $$\begin{aligned} {l_S}(u)& = {} \left\{ {\begin{array}{l} {0,\quad \Leftarrow u \in S;}\\ { + \infty ,\quad \Leftarrow u \notin S.} \end{array}} \right. \nonumber \\ {u_ + }& = {} \max \left\{ {0,u} \right\},\; \log (0) = - \infty ,\quad \hbox {and} \quad 0 \log (0) = 0. \end{aligned}$$

By reformulating TGV as a minimization in the discrete setting, the proposed model can be written as

19 $$\begin{aligned}&\mathop {\min }\limits _u \beta \int \limits _\Omega {(Ku - f\log (Ku))dx} + \frac{\lambda }{2}\left\| u \right\| _2^2+ {\alpha _1}{\left\| {\nabla u - p} \right\| _1}\nonumber \\&\quad + {\alpha _0}{\left\| {\varepsilon (p)} \right\| _1} + {l_{{{\mathbb {R}}^ + }}}(u). \mathrm{{ }} \end{aligned}$$

### The split Bregman algorithm for Poisson noise removal

The split Bregman algorithm (Goldstein and Osher 2009; Wang et al. 2008) has been widely used in image processing, which is easy to be realized and has fast convergence (Cai et al. 2009; Jia et al. 2009). Therefore, we use split Bregman algorithm to solve our minimization problem (19). Firstly, by introducing new auxiliary variables *w*, *x*, *y* and *z*, the problem (19) can be reformulated as the following constrained optimization problem

20 $$\begin{aligned}&\mathop {\min }\limits _u \beta \int _\Omega {w - f\log w} + \frac{\lambda }{2}\left\| u \right\| _2^2 + {\alpha _1}{\left\| x \right\| _1}+ {\alpha _0}{\left\| y \right\| _1}+ {l_{{R^ + }}}(z)\nonumber \\&s.t. \;\; Ku = w, \quad \nabla u - p = x, \quad \varepsilon (p) = y, \quad u = z. \end{aligned}$$

For the above constrained problem (20), we transform it into the corresponding unconstrained problem

21 $$\begin{aligned}&\mathop {\min }\limits _u \beta \int _\Omega {w - f\log w} + \frac{\lambda }{2}\left\| u \right\| _2^2 + {\alpha _1}{\left\| x \right\| _1} + {\alpha _0}{\left\| y \right\| _1}\nonumber \\&\quad + {l_{{R^ + }}}(z) + \frac{{{\mu _1}}}{2}\left\| {Ku - w} \right\| _2^2 + \frac{{{\mu _2}}}{2}\left\| {\nabla u - p - x} \right\| _2^2 \nonumber \\&\quad +\frac{{{\mu _3}}}{2}\left\| {\varepsilon (p) - y} \right\| + \frac{{{\mu _4}}}{2}\left\| {u - z} \right\| _2^2, \end{aligned}$$

where ${\mu _i}(i = 1, \ldots ,4)$ are positive penalty parameters. Thus the split Bregma iterative algorithm for solving the question (20) can be described as

22 $$\begin{aligned}&\left( {{w^{k + 1}},{x^{k + 1}},{y^{k + 1}},{z^{k + 1}},{u^{k + 1}},{p^{k + 1}}} \right) = \mathop {\min }\limits _u \beta \int _\Omega {w - f\log w}\nonumber \\&\quad +\frac{\lambda }{2}\left\| u \right\| _2^2 + {\alpha _1}{\left\| x \right\| _1} + {\alpha _0}{\left\| y \right\| _1} + {l_{{R^ + }}}(z) \mathrm{{ + }}\frac{{{\mu _1}}}{2}\left\| {Ku - w - b_1^k} \right\| _2^2\nonumber \\&\quad +\frac{{{\mu _2}}}{2}\left\| {\nabla u - p - x - b_2^k} \right\| _2^2 + \frac{{{\mu _3}}}{2}\left\| {\varepsilon (p) - y - b_3^k} \right\| _2^2\nonumber \\&\quad +\frac{{{\mu _4}}}{2}\left\| {u - z - b_4^k} \right\| _2^2, \end{aligned}$$

where the updates of the multipliers ${b_1},{b_2},{b_3},{b_4}$ is described as follows

23 $$\begin{aligned} \begin{aligned}&{b_1^{k + 1} = b_1^k + \left( K{u^{k + 1}} - {w^{k + 1}}\right) };\\&{b_2^{k + 1} = b_2^k + \left( \nabla {u^{k + 1}} - {p^{k + 1}} - {x^{k + 1}}\right) };\\&{b_3^{k + 1}= b_3^k + \left( \varepsilon ({p^{k + 1}}) - {y^{k + 1}}\right) };\\&{b_4^{k + 1} = b_4^k + \left( {u^{k + 1}} - {z^{k + 1}}\right) }. \end{aligned} \end{aligned}$$

Since the updates of $b_1^k, b_2^k, b_3^k, b_4^k$ are merely simple calculations, then the minimization question (22) can be divided into the following several subproblems: Given initial value $b_1^0 = b_2^0 = b_3^0 = b_4^0 = 0$ and ${u^0} = {p^0} = {w^0} = {x^0} = {y^0} = {z^0} = 0$, the split Bregman algorithm can be written as

24 $$\begin{aligned} \left\{ \begin{aligned}&{{w^{k + 1}} = \mathop {\arg \min }\limits _w \beta \int _\Omega {w - f\log w} + \frac{{{\mu _1}}}{2}\left\| {K{u^k} - w - b_1^k} \right\| _2^2}, \\&{{x^{k + 1}} = \mathop {\arg \min }\limits _x {\alpha _1}{{\left\| x \right\| }_1} + \frac{{{\mu _2}}}{2}\left\| {D{u^k} - {p^k} - x - b_2^k} \right\| _2^2 },\\&{{y^{k + 1}} = \mathop {\arg \min }\limits _y {\alpha _0}{{\left\| y \right\| }_1} + \frac{{{\mu _3}}}{2}\left\| {\varepsilon ({p^k}) - y - b_3^k} \right\| _2^2 },\\&{{z^{k + 1}} = \max \left\{ {{u^{k + 1}} + b_4^k,0} \right\} },\\&({u^{k + 1}},{p^{k + 1}}) = \mathop {\arg \min }\limits _{u,p} \frac{\lambda }{2}\left\| u \right\| _2^2 + \frac{{\beta {\mu _1}}}{2}\left\| {Ku - {w^{k + 1}} - b_1^k} \right\| _2^2,\\&+ \frac{{{\alpha _1}{\mu _2}}}{2}\left\| {Du - p - {x^{k + 1}} - b_2^k} \right\| _2^2+ \frac{{{\mu _4}}}{2}\left\| {u - {z^{k + 1}} - b_4^k} \right\| _2^2,\\&+ \frac{{{\alpha _0}{\mu _3}}}{2}\left\| {\varepsilon (p) - {y^{k + 1}} - b_3^k} \right\| _2^2. \end{aligned} \right. \end{aligned}$$

For *w*-*subproblem*, note that it is separable with respect to each component. It is easy to solve and the solution of *w* may be written as

25 $$\begin{aligned} {w^{k + 1}} = \frac{1}{2}\left( {\left( {K{u^k} + b_1^k - \frac{\beta }{{{\mu _1}}}} \right) + \sqrt{{{\left( {K{u^k} + b_1^k - \frac{\beta }{{{\mu _1}}}} \right) }^2} + \frac{{4\beta f}}{{{\mu _1}}}} } \right) . \end{aligned}$$

As for solving *x*, *y*-*subproblem*, we can directly obtain the solutions by using shrinkage operator:

The *x*-*subproblem* can be solved by

26 $$\begin{aligned} {x^{k + 1}} = \max \left( {\left\| {D{u^k} - {p^k} - b_2^k} \right\| _2} - \frac{{{\alpha _1}}}{{{\mu _2}}},0 \right) \frac{{D{u^k} - {p^k} - b_2^k}}{{{{\left\| {D{u^k} - {p^k} - b_2^k} \right\| }_2}}}. \end{aligned}$$

The solution of the *y*-*subproblem* is similarly obtained

27 $$\begin{aligned} {y^{k + 1}}\! = \max \left( {\left\| {\varepsilon ({p^k}) - b_3^k} \right\| _2} \!- \frac{{{\alpha _0}}}{{{\mu _3}}},0 \right) \frac{{\varepsilon ({p^k}) \!- b_3^k}}{{{{\left\| {\varepsilon ({p^k}) \!- b_3^k} \right\| }_2}}}. \end{aligned}$$

The (*u*, *p*)-subproblem is a saddle-point problem, which can be divided into the following two subproblems:

1. For *u*, we have
  28 $$\begin{aligned} \begin{aligned} {u^{k + 1}}&= \mathop {\arg \min }\limits _u \frac{\lambda }{2}\left\| u \right\| _2^2 + \frac{{\beta {\mu _1}}}{2}\left\| {Ku - {w^{k + 1}} - b_1^k} \right\| _2^2\\&\quad + \frac{{{\alpha _1}{\mu _2}}}{2}\left\| {Du - p - {x^{k + 1}} - b_2^k} \right\| _2^2 + \frac{{{\mu _4}}}{2}\left\| {u - {z^{k + 1}} - b_4^k} \right\| _2^2, \end{aligned} \end{aligned}$$
  which can be solved by considering the following normal equation
  29 $$\begin{aligned} \begin{aligned}&\lambda u + {\alpha _1}{\mu _2}\sum \limits _{j = 1}^2 {D_j^T\left( {{D_j}u - {p_j} - x_j^{k + 1} - b_{{2_j}}^k} \right) } \\& \quad + \beta {\mu _1}{K^T}\left( {Ku - {w^{k + 1}} - b_1^k} \right) + {\mu _4}\left( {u - {z^{k + 1}} - b_4^k} \right) = 0. \end{aligned} \end{aligned}$$
  Finally, *u* is solved by
  $$\begin{aligned} \begin{aligned} {u^{k + 1}}&= {\left( \lambda I + \beta {\mu _1}{K^T}K + {\alpha _1}{\mu _2}\sum \limits _{j = 1}^2 {D_j^T{D_j}} + {\mu _4}I \right) ^{ - 1}}\\&\quad \times \left( \beta {\mu _1}{K^T}\left( {w^{k + 1}} + b_1^k \right) \right. + {\alpha _1}{\mu _2}\sum \limits _{j = 1}^2 {D_j^T} \left( {p_j} + x_j^{k + 1} + b_{{2_j}}^k \right) \\&\quad \left. {+ {\mu _4}({z^{k + 1}} + b_4^k)} \right) . \end{aligned} \end{aligned}$$
2. For the sub-problem *p*, it can be written as the following minimization problem
  30 $$\begin{aligned} \begin{aligned} {p^{k + 1}}& = {} \mathop {\arg \min }\limits _p \frac{{{\alpha _1}{\mu _2}}}{2}\left\| {Du - p - {x^{k + 1}} - b_2^k} \right\| _2^2\\& \quad + \frac{{{\alpha _0}{\mu _3}}}{2}\left\| {\varepsilon (p) - {y^{k + 1}} - b_3^k} \right\| _2^2, \end{aligned} \end{aligned}$$
  where $p=(p_1,p_2)^T$ is a $2\times 1$ vector, $\varepsilon (p)$ is a $2\times 2$ matrix.
  For $p_1$, it can be solved by considering the following linear system
  31 $$\begin{aligned}&{\alpha _1}{\mu _2}\left( {{p_1} - {D_1}u + x_1^{k + 1} + b_{{2_1}}^k} \right) + {\alpha _0}{\mu _3}D_1^T\left( {{D_1}{p_1} - y_1^{k + 1} - b_{{3_1}}^k} \right) \nonumber \\&\quad +\frac{{{\alpha _0}{\mu _3}}}{2}D_2^T\left( {{D_2}{p_1} + {D_1}{p_2} - 2y_3^{k + 1} - 2b_{{3_3}}^k} \right) = 0. \end{aligned}$$
  Therefore,
  32 $$\begin{aligned} p_1^{k + 1}& = {({\alpha _1}{\mu _2}I + {\alpha _0}{\mu _3}D_1^T{D_1} + \frac{{{\alpha _0}{\mu _3}}}{2}D_2^T{D_2})^{ - 1}} \nonumber \\& \quad \times ({\alpha _1}{\mu _2}({D_1}u - x_1^{k + 1} - b_{{2_1}}^k) + {\alpha _0}{\mu _3}D_1^T(y_1^{k + 1} + b_{{3_1}}^k)\nonumber \\& \quad +\frac{{{\alpha _0}{\mu _3}}}{2}D_2^T(2y_3^{k + 1} + 2b_{{3_3}}^k - {D_1}{p_2})). \end{aligned}$$
  Similarly, we can obtain the solution of $p_2$ as
  33 $$\begin{aligned} p_2^{k + 1}& = {\left( {\alpha _1}{\mu _2}I + {\alpha _0}{\mu _3}D_2^T{D_2} + \frac{{{\alpha _0}{\mu _3}}}{2}D_1^T{D_1}\right) ^{ - 1}} \nonumber \\& \quad \times {\alpha _1}{\mu _2}\left( {D_2}u - x_2^{k + 1} - b_{{2_3}}^k \right) + {\alpha _0}{\mu _3}D_2^T\left( y_2^{k + 1} + b_{{3_2}}^k \right) \nonumber \\& \quad + \frac{{{\alpha _0}{\mu _3}}}{2}D_1^T\left( 2y_3^{k + 1} + 2b_{{3_3}}^k - {D_2}{p_1}\right) . \end{aligned}$$

## Experimental results and discussions

In this section, we illustrate some numerical results of the proposed model for the Poisson noise removal problem. We compare our method with the one proposed in Figueiredo and Bioucas-Dias (2010) (PIDAL) and the other proposed in Liu and Huang (2012) (PID-Split). In order to prove the superiority of the proposed model, we compare our model with TGV regularization model.To show the effectivity of the proposed model, we choose four pictures possed abundant detail information.

We terminate the iterations for these methods by the following stopping criterion

34 $$\begin{aligned} \frac{{{{\left\| {{u^{k + 1}} - {u^k}} \right\| }_2}}}{{{{\left\| {{u^k}} \right\| }_2}}} \le 1 \times {10^{ - 3}}. \end{aligned}$$

The quality of the restoration results is compared quantitatively by using the Signal-to-Noise Ratio (SNR), the Peak Signal-to-Noise (PSNR), the relative error (RelErr) and the Structural SIMilarity index (SSIM). They are defined as follows

35 $$\begin{aligned} \mathrm{Re} lErr& = {} \frac{{{\left\| u - \widehat{u} \right\| }_2}}{{{{\left\| u \right\| }^2}}}, \;\;SNR = 20{\log _{10}}\left( \frac{{{{\left\| u \right\| }_2}}}{{{{\left\| {\widehat{u} - u} \right\| }_2}}}\right) , \end{aligned}$$

36 $$\begin{aligned} MSE& = {} \frac{1}{{\left| \Omega \right| }}\int _\Omega {{{\left( {\widehat{u} - u} \right) }^2}dx},\;\;PSNR = 10{\log _{10}}\left( \frac{{{{255}^2}}}{{MSE}}\right) , \end{aligned}$$

where *u* and $\widehat{u}$ are the ideal image and the restored image, respectively.

37 $$\begin{aligned} SSIM = \frac{{\left( {2{\mu _u}{\mu _{\widehat{u}}} + {C_1}} \right) \left( {2{\sigma _{u\widehat{u}}} + {C_2}} \right) }}{{\left( {\mu _u^2 + \mu _{\widehat{u}}^2 + {C_1}} \right) \left( {\sigma _u^2 + \sigma _{\widehat{u}}^2 + {C_2}} \right) }}, \end{aligned}$$

where $\mu _u$ and $\mu _{\widehat{u}}$ are averages of *u* and $\widehat{u}$, respectively. $\sigma _u$ and $\sigma _{\widehat{u}}$ are the variance of *u* and $\widehat{u}$, respectively. $\sigma _{u{\widehat{u}}}$ is the covariance of *u* and $\widehat{u}$. The positive constants $C_1$ and $C_2$ can be thought of as stabilizing constants for near-zero denominator values. Generally speaking, the more bigger value of SNR, PSNR or the smaller value of RelErr is, the better quality of the reconstructed image is.

The Poissonian images used for our experiments are generated as follows: the original images are convoluted with a blur kernel and additionally contaminated by Poisson noise, here we use the $\mathbf {poissrnd}$ function in MATLAB’s Statistics Toolbox after blurring the true images with the given point spread functions to generate the blurred and noise images.

The selection of the regularization parameters highly affects the image restoration results, and related to make the fair comparison with different denoising models. The penalty parameters $\mu$ which relies on unknown noise level highly influences the speed of the algorithms. In experiments, we set $\mu =[0.01, 0.001]$ in the PIDAL algorithm. In the PID-Split algorithms, we choose $\mu =[0.0004; 0.1, 0.0001]$. In the TGV model, we set $\mu =[0.1; 10, 5, 3]$. The penalty parameter in the proposed method is empirically set $\mu =[0.1; 0.6; 0.1; 0.02]$. Thus, we may have a good restoration results.

**Fig. 1:**
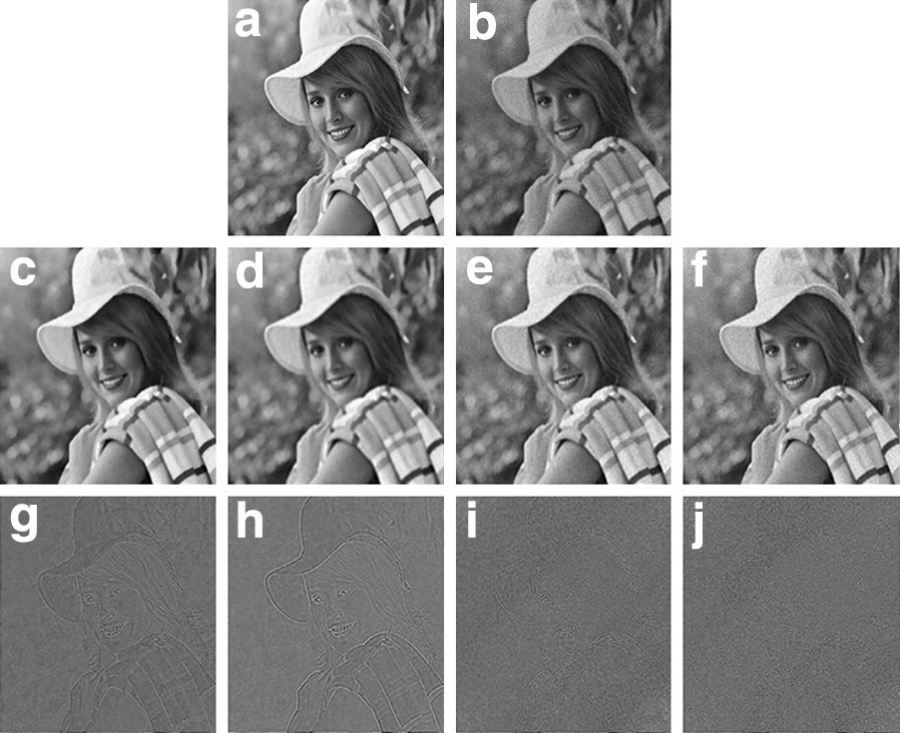
The woman picture is compared with other method. **a** Original image; **b** degraded image; **c** the PIDAL model result; **d** the PID-Split method result; **e** the TGV method result; **f** the proposed model result. **g**–**j** The residual images

In the first experiment, we used the image “Woman” ($512\times 512$) in Fig. 1a. We perform the blurring operation *psfGauss*(5, 2) proposed in Nagy et al. (2004) on the original image and add the Poisson noise to the blurred data to generate the degraded image in Fig. 1b. The parameter of this test, we set $\beta =120$ in PIDAL algorithm, $\beta =6,\lambda =0.01$ for PID-Split algorithms,due to the TGV model we set $\beta =450, \alpha =[8, 10]$, set $\beta =54, \lambda =0.001, \alpha =[16, 9]$ for the proposed model. The pictures of Fig. 1c–f are the restoration images, which represent the difference between the three methods. From these pictures, we can see the proposed model have more advantages. In order to more effectively reflect the experiment result, Fig. 1g–j present the residual images refer to the difference of the original image and the restoration image. From these pictures, we can see that the proposed model can preserve more details than other methods. In the Table 1, the *SNR*, *PSNR*, *RelErr* and *SSIM* values of the restored images by the proposed model are better than other methods.

**Fig. 2:**
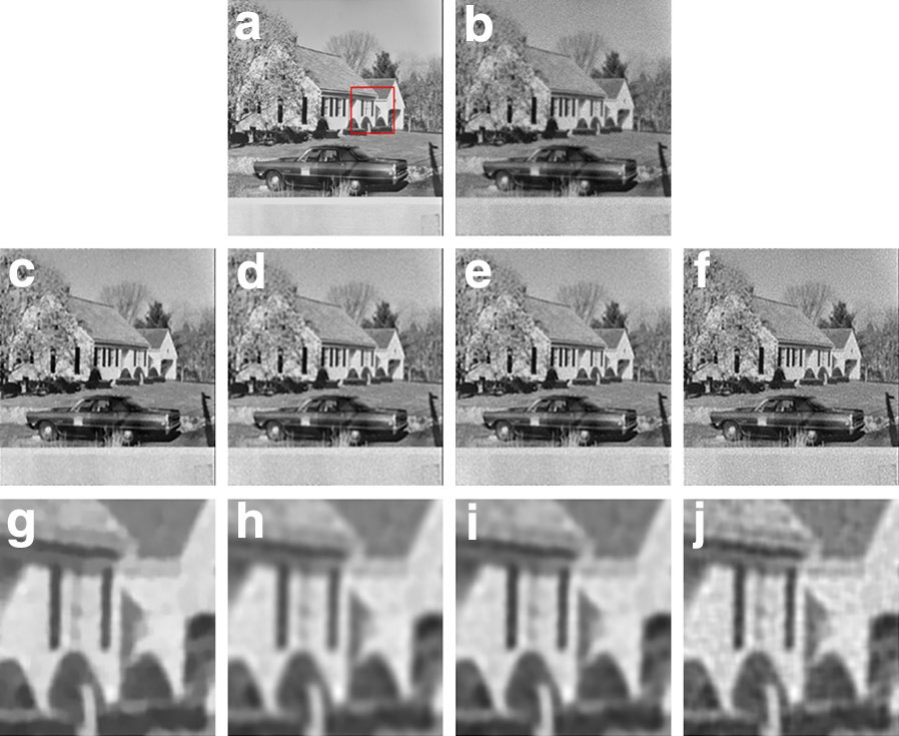
The house picture is compared with other method. **a** Original image; **b** degraded image; **c** the PIDAL model result; **d** the PID-Split method result; **e** the TGV method result; **f** the proposed model result; **g**–**j** The result of partial enlarged pictures

In second experiment, we use the image “House” with size ($512\times 512$) in Fig. 2a, which contains a lot of edge details. We perform the blurring operation with radius 5 ($psf = ones(5,5)/25$) on the original image and add the Poisson noise to the blurred data to generate the degraded image in Fig. 2b. As for parameter selection, we choose $\beta =200$ for the PIDAL algorithm, $\beta =20, \lambda =0.00001$ for the PID-Split algorithm, set $\beta =450, \alpha =[8, 10]$ in the TGV model. The proposed algorithm we set $\beta =75, \lambda =0.00001, \alpha =[17, 13]$. Form Fig. 2c–f, we can see that the proposed model compared to the *PIDAL* method and PID-Split algorithms have better restoration results. In Fig. 2g–j, we have enlarged some details of the images, which can be clearly see the advantages of the proposed model for the recovery of edge details. The *SNR*, *RelRrr* and *SSIM* values in Table 1 showed that the proposed model have a better restoration result.

**Fig. 3:**
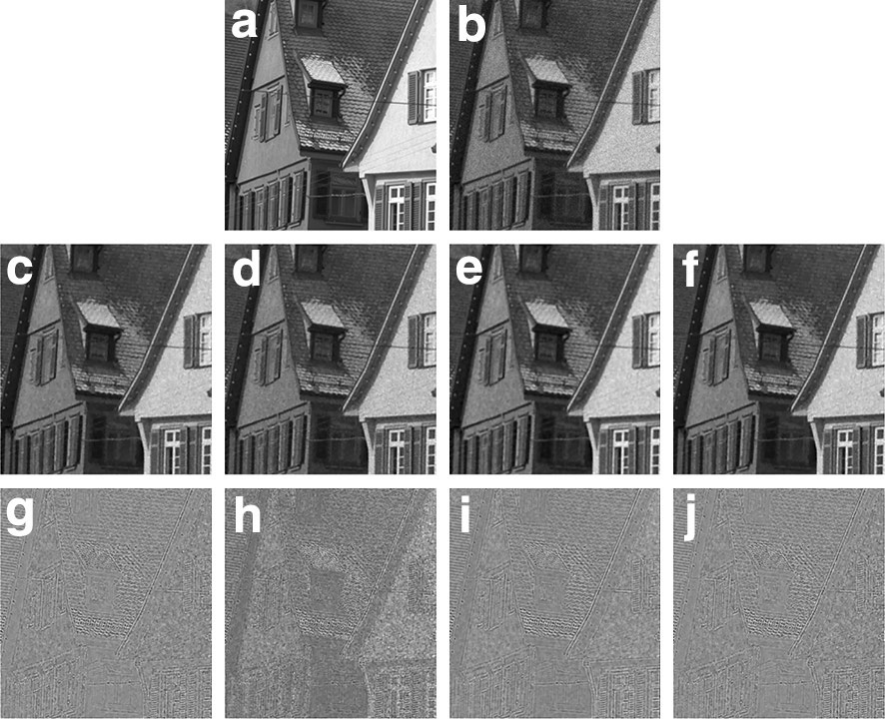
The third experiment. **a** Original image; **b** degraded image; **c** the PIDAL model result; **d** the PID-Split method result; **e** the TGV method result; **f** the proposed model result; **g**–**j** The residual images

In order to further verify the validity of the new model, in third experiment, we use the image which contains a lot of edge details. We perform the blurring operation by a line motion blur. The point spread function for the linear motion blur is returns a filter to approximate, once convolved with an image, the linear motion of a camera by *r* pixels, with an angle of $\theta$ degrees in a counter-clockwise direction. In this example, $r = 2$ and $\theta = 45$, then add the Poisson noise to the blurred data to generate the degraded image in Fig. 3b. The parameters choose as the same as those in second experiment and also may be adjusted.

**Table 1:** Summarized all of the experiment restoration results

Method	SNR	PSNR	RelErr	SSIM
Test1				
PIDAL (Figueiredo and Bioucas-Dias 2010)	25.33	30.30	0.054	0.904
PID-Split (Liu and Huang 2012)	25.18	30.16	0.055	0.917
TGV	25.31	30.32	0.053	0.908
Proposed	25.48	30.45	0.053	0.922
Test2				
PIDAL (Figueiredo and Bioucas-Dias 2010)	21.17	25.03	0.087	0.828
PID-Split (Liu and Huang 2012)	21.38	25.36	0.084	0.837
TGV	21.24	25.10	0.086	0.835
Proposed	21.74	25.60	0.080	0.843
Test3				
PIDAL (Figueiredo and Bioucas-Dias 2010)	21.50	27.87	0.0841	0.811
PID-Split (Liu and Huang 2012)	21.49	27.86	0.0357	0.811
TGV	20.64	27.01	0.092	0.801
Proposed	21.01	27.23	0.0803	0.820

**Fig. 4:**
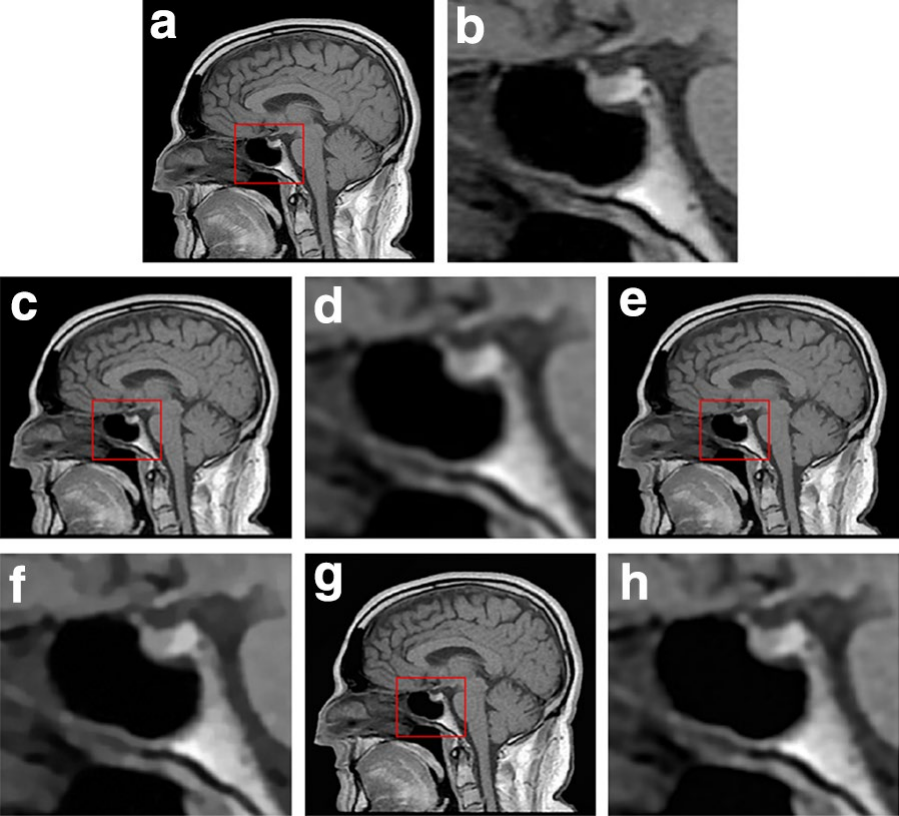
The fourth experiment. **a** Ground truth; **b** ground truth enlarge; **c** PID-Split method result; **d** PID-Split method enlarge; **e** PIDAL-Method result; **f** PIDAL-method enlarge; **g** Proposed method result; **h** proposed method enlarge

Finally, let us choose a human brain MR image of size $240\times 240$ as the test image. We zoom in a marked close-up region which is abundant in texture-like features to better visual comparison. We can clearly see that the produced textures by our proposed method are better quality than the other methods from Fig. 4.

## Conclusions

In this paper, we investigate the second-order total generalized variation with a quadratic regularization to deal with the Poissonian images restoration problem. The proposed model is solved efficiently by split Bregman iterative algorithm in this way the calculation speed is fast. Numerical results show that our proposed method is particularly advantageous for restoration the Poisson images in terms of *SNR*, *SSIM* and *RelErr* quality compared to other methods. In the model, the parameters selection is a difficult problem which needs further study.
